# Role of Macrophages in Air Pollution Exposure Related Asthma

**DOI:** 10.3390/ijms232012337

**Published:** 2022-10-15

**Authors:** Chung-Hsiang Li, Mei-Lan Tsai, Hsin-Ying (Clair) Chiou, Yi-Ching Lin, Wei-Ting Liao, Chih-Hsing Hung

**Affiliations:** 1 Department of Pediatrics, Kaohsiung Medical University Hospital, Kaohsiung Medical University, Kaohsiung 807, Taiwan; 2 Department of Pediatrics, Kaohsiung Municipal Ta-Tung Hospital, Kaohsiung 801, Taiwan; 3 Graduate Institute of Medicine, College of Medicine, Kaohsiung Medical University, Kaohsiung 807, Taiwan; 4 Department of Pediatrics, Faculty of Pediatrics, College of Medicine, Kaohsiung Medical University, Kaohsiung 807, Taiwan; 5 Teaching and Research Center of Kaohsiung Municipal Siaogang Hospital, Kaohsiung 812, Taiwan; 6 Department of Laboratory Medicine, Kaohsiung Medical University Hospital, Kaohsiung Medical University, Kaohsiung 807, Taiwan; 7 Department of Laboratory Medicine, School of Medicine, College of Medicine, Kaohsiung Medical University, Kaohsiung 807, Taiwan; 8 Doctoral Degree Program in Toxicology, College of Pharmacy, Kaohsiung Medical University, Kaohsiung 807, Taiwan; 9 Department of Medical Research, Kaohsiung Medical University Hospital, Kaohsiung Medical University, Kaohsiung 807, Taiwan; 10 Department of Biotechnology, College of Life Science, Kaohsiung Medical University, Kaohsiung 807, Taiwan; 11 Research Center for Environmental Medicine, Kaohsiung Medical University, Kaohsiung 807, Taiwan; 12 Department of Pediatrics, Kaohsiung Municipal Siaogang Hospital, Kaohsiung 812, Taiwan

**Keywords:** asthma, air pollution, particulate matter, heavy metals, macrophages

## Abstract

Asthma is a chronic inflammatory airway disease characterized by variable airflow obstruction, bronchial hyper-responsiveness, and airway inflammation. The chronic inflammation of the airway is mediated by many cell types, cytokines, chemokines, and inflammatory mediators. Research suggests that exposure to air pollution has a negative impact on asthma outcomes in adult and pediatric populations. Air pollution is one of the greatest environmental risks to health, and it impacts the lungs’ innate and adaptive defense systems. A major pollutant in the air is particulate matter (PM), a complex component composed of elemental carbon and heavy metals. According to the WHO, 99% of people live in air pollution where air quality levels are lower than the WHO air quality guidelines. This suggests that the effect of air pollution exposure on asthma is a crucial health issue worldwide. Macrophages are essential in recognizing and processing any inhaled foreign material, such as PM. Alveolar macrophages are one of the predominant cell types that process and remove inhaled PM by secreting proinflammatory mediators from the lung. This review focuses on macrophages and their role in orchestrating the inflammatory responses induced by exposure to air pollutants in asthma.

## 1. Introduction

Chronic respiratory diseases affect approximately 45 million people worldwide. Asthma is one of the largest contributors to global respiratory disease and affects about 21 million people [1]. The characteristics of asthma include recurrent symptoms of wheezing, cough, chest tightness, and dyspnea. Epidemiology evidence suggests that environmental exposure, especially to inhaled agents such as allergens, pollutants, and viruses, provokes and exacerbates asthma attacks.

Air pollution is a significant health risk factor and is associated with a high risk for premature death due to cardiovascular diseases (e.g., ischemic heart disease, chronic obstructive pulmonary disease, asthma, lower respiratory infections, and lung cancer). The WHO reported that exposure to outdoor air pollution caused 4.2 million premature deaths worldwide in 2016 [2]. Ambient air pollution is classically composed of particulate matter (PM) and several gases, including ozone (O_3_), volatile organic compounds (VOCs), carbon monoxide (CO), and nitrogen oxides (NOx). PM is a major component of air pollution, composed mainly of organic and elemental carbon, metals, and polycyclic aromatic hydrocarbons, and has well-documented associations with serious short- and long-term adverse health effects [3]. PM can be further categorized into three groups according to the aerodynamic equivalent diameter of the particles as follows: PM10 (coarse particles between 2.5 to 10 µm), PM2.5 (fine particles smaller than 2.5 µm), and ultrafine particles (UFP, smaller than 0.1 µm). The production of PM10 particles is mainly from the suspension of disturbed soils, road and industrial dust, and construction debris, while PM2.5 and UFP are primarily derived from direct emissions from the combustion processes of coal, oil, and gasoline burning and the transformation gas products of NO with SO_2_ [4]. Previous studies also showed that reduced lung function, the incidence and exacerbation of chronic respiratory diseases, and an elevated admission rate were associated with either short-term or long-term exposure to PM [5,6]. It has been proven that the adverse health effects of PM are directly linked to the size of PM [4]. Particles exceeding 10 μm in diameter are less likely to enter the lower respiratory tract, as they are mostly eliminated by the cilia and mucus in the nose and upper airway, whereas particles less than 10 μm in diameter are deposited deeply in the lower respiratory tract. Therefore, they have the most influence on human health [7].

Macrophages are widely present in the body and are commonly used to maintain homeostasis and resist pathogen invasion. According to changes in their environment, macrophages are polarized into different macrophage subtypes, such as M1 macrophages and M2 macrophages [8]. M1 macrophages are capable of proinflammatory responses and produce proinflammatory cytokines. In contrast, M2 macrophages are capable of anti-inflammatory responses and repair damaged tissues [9]. Since macrophages play an important role in regulating immune responses and metabolism, macrophage polarization disorder may cause some diseases. A role for macrophages, in particular, has long remained understudied. However, it is now known that they can polarize into many different phenotypes and that these differentially polarized phenotypes are changed in asthma [10,11]. The inhalation of air pollution particles induces an immune response initiated by alveolar macrophages (AMs) and airway epithelial cells in the lungs. Macrophages are several times more potent in producing proinflammatory mediators that contribute to the local inflammatory response in the lung and the subsequent systemic inflammatory response [12]. Therefore, understanding the functional heterogeneity and ontogeny of lung macrophages should help develop future macrophage-based therapies for human chronic respiratory diseases.

## 2. Origins of Lung Macrophages

Macrophages are the first immune cells to appear in an organism’s development and are essential during the early stages of development [13]. In the lung, there are two types of macrophages, interstitial macrophages (IMs) and alveolar macrophages (AMs), which reside in different anatomical compartments [14]. AMs reside on the epithelial surface, in direct contact with the environment, and are critical sentinels of lung function and barrier immunity. They prevent tissue damage by phagocytosing pathogens and initiate protective immune responses [15]. The colonization of AMs depends on the yolk sac, fetal liver, or bone marrow and efficient macrophage function [16,17,18]. AMs normally live independent of blood monocyte input. When AMs are damaged or depleted, new macrophages are recruited from circulation and contribute to repopulating the AM niche [19]. IMs are one uniform cell population that resides in the interstitium and are less well studied than AMs [20]. According to cell surface markers, the macrophage populations in the lung are defined as AMs, IMs, and monocyte-derived cells. AMs are recognized by high side scatter, high expression of CD206, and relatively low expression of the monocyte marker CD14. Unlike AMs, IMs are negative for CD169 (Siglec-1 or sialoadhesin), a cell adhesion molecule shown to discriminate between AM (CD206^+^CD169^+^) and IM (CD206^+^CD169^−^) phenotypes [21]. There are three subpopulations of IMs, including IM1 (MHCII^low^CD206^high^), IM2 (MHCII^+^CD206^high^), and IM3 (MHCII^high^CD206^low^CCR2^+^). A few studies have demonstrated that the major function of IMs is to maintain immune homeostasis and induce immune tolerance to benign antigens [22]. In addition to the population of AMs and IMs, CD14^+^ monocyte-derived cells are another population in the lungs. By expressing CD206, CD141, CD11c, human leukocyte antigen DR (HLA-DR), and CC-chemokine receptor type 7 (CCR7), these cells can be distinguished from intravascular CD14^+^ monocytes [23]. AMs are the major effector cells of immune responses and participate in pro- and anti-inflammatory functions.

## 3. Macrophage Polarization in Asthma

Asthma is a common, complex respiratory disease that is characterized by recurrent symptoms of wheeze, cough, chest tightness, and dyspnea. There is growing evidence of environmental triggers that exacerbate the disease, especially inhaled agents such as allergens, pollutants, and viruses [24]. Depending on the microenvironment of the lung, macrophages can change their gene expression to two polarized macrophage profiles, M1 (proinflammatory) and M2 (anti-inflammatory) [25]. These cells play important roles in the protection against various respiratory pathogens, partly by tightly regulated NLRP3 inflammasome-dependent mechanisms [26]. In animal models, it was revealed that the mostly immunosuppressive function of AMs in airway allergies is partially mediated by CD40, TNF-α, PGE2, and the secretion of exosomes and may be defective in asthmatic patients [27,28]. The depletion of resident AMs led to significant worsening of allergic airway inflammation in mice [29]. The bronchial macrophages in asthmatic patients expressed less CD16 and CD64 and showed decreased phagocytic activities and anti-inflammatory capabilities [30]. The activated M2 macrophages were proposed to mediate type 2 immunity. However, their exact contribution to disease pathogenesis is still under investigation [31]. Importantly, some experiments also demonstrated a proinflammatory role for AMs in the context of allergic airway inflammation. In mouse models, AMs were found to be significant sources of IL-17, and their depletion prevented the development of allergic inflammation and airway hyper-responsiveness [32]. Moreover, we previously used a new macrophage marker, PM-2K, to analyze the association of macrophage polarization in the peripheral blood with the severity of adult and childhood asthma. We found that PM-2K+ macrophages were significantly lower, particularly the CD14^−^PM-2K^+^ subset and M2b (PM-2K^+^CCR7^−^CD86^+^) cells, in subjects with poor lung function compared to normal subjects and asthmatics with normal lung function, whereas the frequency of fibrocytes was higher in asthmatics, and the M2b subset distributions were significantly different in subjects with varying severities [33]. In contrast, the PM-2K^+^CD14^+^ but not PM-2K^+^CD14^−^ was lower in asthmatic children than in healthy children. In asthmatic children, the M2a (CCR7^−^CXCR1^+^), M2b (CCR7^−^CD86^+^), and M2c (CCR7^−^CCR2^+^) subsets, but not M1 (CCR7^+^CD86^+^), were higher in asthmatic children than in healthy children. Significantly, the percentage of the M2c subset was positively associated with asthmatic children requiring hospitalization during exacerbations. It was demonstrated that macrophage polarization is involved in the pathogenesis of adult and childhood asthma and is a potential biomarker of asthma disease severity [34].

## 4. Initial Process Reactions after Introducing Particulate Matter to Macrophages

Alveolar macrophages are important in the regulation of innate immune responses against invading pathogens, the initiation of an inflammatory response, tissue repairing, and further initiating an adaptive immune response. Alveolar macrophages remove the invading microorganisms, tissue, and environmental debris in the lungs via phagocytosis; in addition, the clearance of apoptotic cells through efferocytosis in macrophages maintains homeostasis in the lungs, which was well-demonstrated in the previous literature [35,36]. The alterations of macrophage-related cytokines after PM stimulation are shown in Table 1.

### 4.1. Phagocytosis

Alveolar macrophages decrease the inflammatory stimuli in the respiratory tract by removing and processing pathogens with debris via phagocytosis. However, the ability of macrophage phagocytosis is reduced by exposure to air pollutants. Therefore, carbon loading in airway macrophages has been proposed as a potential biomarker for PM exposure [53,54]. Morrow demonstrated that under chronic dust inhalation stimulation PM-laden alveolar macrophages showed impaired function, including decreased motility and mucociliary clearance ability [55]. In a cross-sectional study in healthy children, the carbon content of airway macrophages was inversely associated with lung function status [56]. In addition, previous studies revealed that exposure to PM resulted in decreased macrophage phagocytosis in both murine and human models [50,57,58,59]. PM2.5 exposure to pneumococcus-infected mice led to macrophage dysfunctions, including impairing phagocytosis with nitric oxide production; in addition, decreased bacterial clearance activity via dampened proinflammatory cytokine expression was also observed. Therefore, PM exposure promotes the infectivity of pneumococcus and contributes to further pulmonary inflammation [60].

### 4.2. Efferocytosis

Macrophages also play an important role in the clearance of apoptotic cells via efferocytosis, a process that prevents secondary necrosis and the release of proinflammatory cytokines, which results in the resolution of lung inflammation [61]. A previous study demonstrated that impaired macrophage efferocytosis function was found in asthma patients [62]. Furthermore, a recent murine model study revealed that exposure to air pollutants resulted in decreased macrophage efferocytosis [63]. In another murine model, exposure to ozone resulted in reduced macrophage efferocytosis compared with exposure to filtered air [64].

### 4.3. Autophagy

Autophagy is a self-degradative process that consists of the formation of autolysosomes to remove damaged organelles, cell debris, and intracellular pathogens to achieve homeostasis [65]. Previous research has shown that autophagic defects may play a critical role in nanomaterial toxicity [66]. Exposure to fine PM (FPM) in human macrophages could inhibit phagocytosis by promoting the expression of autophagy-related 2A (ATG2A) and reactive oxygen species generation via the extracellular signal-regulated kinase 1/2 (ERK1/2) signaling pathway, which leads to further autophagy dysfunction. Moreover, FPM compromised the phagocytic ability of macrophages on Escherichia coli and apoptotic neutrophils [59].

### 4.4. Apoptosis

In addition to reducing macrophage function, exposure to PM also promotes macrophage apoptosis in both mouse and human models [42,67]. It has been shown that the surface component of PM is responsible for promoting alveolar macrophage apoptosis via binding to scavenger receptor class A in a mouse model [68]. Exposure to fine PM triggered rat macrophage apoptosis via mitochondria-mediated apoptotic mechanisms (by both decreasing the expression of Bcl-2 and increasing the expression of Bax) and increased oxidative stress involving the production of NO and ROS to promote further lung inflammation [69]. While the current evidence supports that exposure to air pollutants promotes lung inflammation by reducing macrophage phagocytosis with efferocytosis and promoting macrophage apoptosis, the exact mechanisms still need further investigation.

## 5. Immune Reaction after Exposure to Particulate Matter

To eliminate the threat of air pollutant components, macrophages provoke inflammatory reactions by elaborating cytokines and directly maintain homeostasis by phagocytosis with efferocytosis. Macrophages can recognize foreign air pollutants via several cellular mechanisms, including Toll-like receptors (TLRs), reactive oxygen species (ROS) pathways, and polyaromatic hydrocarbon (PAH) pathways. After being recognized by these pathways, downstream intracellular signaling, such as the NFkB and MAPK pathways, are activated to promote proinflammatory responses [70].

### 5.1. Toll-like Receptor (TLR) Responses

TLRs are a cluster of cellular receptors that aim to recognize pathogen-associated molecular patterns (PAMPs) and damage-associated molecular peptides (DAMPs) to prevent the invasion of foreign pathogens and other stimuli such as air pollutants. PM can be recognized by TLRs either by the microorganism contents such as lipopolysaccharide (LPS) and fungal spores or host-derived molecules such as oxidized phospholipids with nucleic acids from damaged cells [71]. Exposure to air pollutants provokes TLR expression and the further activation of macrophage response through PAMPs with DAMPs, a process known as priming [72]. Previous animal experimental studies have proven that exposure to air pollutants can provoke proinflammatory cytokine release via priming [52], and this process predominantly involves the TLR4 [43,44,63,73,74,75,76] and TLR2 [76,77] signaling pathways. Exposure to inhaled ozone results in increased expression of TLR4 and CD14 as well as the proinflammatory cytokine tumor necrosis factor-alpha (TNF-α) in healthy human macrophages [78]. In addition, exposure to air pollutants in murine macrophages also alters the interactions between the TLR4 and TLR5 pathways. TLR5 could promote TLR4-mediated inflammation by enhancing the MyD88-dependent pathway [79].

### 5.2. Reactive Oxygen Species (ROS) Reaction

It has been proven that PM exposure causes the direct generation of oxidants (reactive oxygen species) and a reduction in endogenous antioxidants, which leads to increased oxidative stress [80]. This stimulation of oxidative stress by PM contributes to both its organic (donates electrons to form superoxide free radicals) and metal compounds (donates electrons to form superoxide with hydrogen peroxide and depletes endogenous antioxidants). Oxidative stress after PM exposure initiates cellular responses, including the activation of the mitogen-activated protein (MAP) kinase cascade (including ERK, p38, and Jun kinases) and NF-κB signaling to enhance the production of inflammatory cytokines, which causes further cell injury or apoptosis [80].

Furthermore, oxidative stress by PM exposure not only stimulates NF-κB and AP-1 signaling but also increases the transcription of genes containing the antioxidant-responsive element (ARE) promoter, although the specific mechanisms remain unclear [81]. By activating reactive oxygen species, PM initiates intracellular danger signals to induce NLRP3 inflammasome (including NLRP3 protein and inactive caspase-1) responses. Activating caspase-1 leads to the cleavage of immature prointerleukin (IL)-1β to mature active IL-1β, which contributes to further proinflammatory responses in epithelial cells [82]. NLRP3 inflammasome responses after exposure to PM were proven in both mouse [49] and human [83] models.

### 5.3. Polyaromatic Hydrocarbon (PAH)

Oxidative stress can also be induced by polyaromatic hydrocarbon molecules on PM, which can be sensed by the aryl hydrocarbon receptors (AhRs) in cells. The AhR is a cytosolic receptor that is widely spread in the body and is involved in sensing environmental alterations, for example, circadian rhythm or oxygen gradients. Binding AhR with PAH ligands activates xenobiotic responses, including enhanced CYP1A1 and CYP1B1 enzyme production, which leads to the production of cytotoxic or genotoxic products [84]. The introduction of urban PM to human monocytes resulted in increased TNF-α expression and secretion; in addition, PM also increased monocyte CYP1A1 expression via AhR [85]. In another mouse model, ultrafine-particle-associated PAHs aggravated allergic airway inflammation by inducing an AhR-dependent induction of Jagged 1 (Jag1) expression in lung AMs and led to Th2 and Th17 cell differentiation in allergen-specific T cells [86]. In addition, PAHs binding with AhR can cross talk with other inflammatory and antioxidant transcription factors, including RORγt, STAT1, Nrf2, and NFκB [84], which implies an important role of PAH within PM in lung inflammatory homeostasis.

## 6. Inflammatory Mediator Production after Exposure to Particulate Matter

### 6.1. Proinflammatory Cytokines

It is well-known that exposure to PM in alveolar macrophages induces the production of the proinflammatory cytokines, especially TNF-α, IL-1β, IL-6, and IL-8, in both murine [42,46,52] and human [38,87,88] models. In an animal model, alveolar macrophages produced more TNF-α, IL-1β, and IL-6 than bronchial epithelial cells after being exposed to the same dosage of PM, which suggests an important role of alveolar macrophages in initiating proinflammatory responses after PM stimulation [89]. Moreover, synergistic effects between human macrophages and epithelial cells in proinflammatory responses were reported by Fujii et al. [90] and Ishii et al. [91]. After PM10 stimulation, proinflammatory cytokines such as IL-6 and granulocyte-macrophage colony-stimulating factor (GM-CSF) in AM/epithelial cell cocultures were significantly increased compared to epithelial cells or AMs alone. Furthermore, previous in vitro animal [46] and human [51] studies have shown that larger particles are more potent to trigger stronger proinflammatory responses; coarse particles (2.5–10 μm) stimulated the production of more proinflammatory cytokines such as TNF-α, IL-6, and IL-8 than fine particles (<2.5 μm), which may be related to a higher level of particulate endotoxin content in coarse particles.

### 6.2. The Effect of PM Exposure on Macrophage Polarization

The effect of PM on alveolar macrophage polarization remains inconclusive [71]. As mentioned above, PM exposure could promote M1 cytokine (IL-12 and interferon-gamma, IFN-r) expression and suppress M2 cytokine (such as IL-4, IL-5, IL-13, and IL-10) production [42,90], which implies that PM exposure induced a trend toward M1 differentiation [92]. However, recent animal studies revealed opposite conclusions that prolonged PM exposure may also promote M2 cytokine expression, which leads to allergic responses in the respiratory tract. Furthermore, continuous airway PM2.5 exposure resulted in increased eosinophils and macrophages in bronchial alveolar lavage fluid; proinflammatory cytokines (such as IL-1β, monocyte chemoattractant protein MCP-1, and IL-12) and M2 cytokines (including IL-5, IL-13, and PGD2) were also elevated significantly. PM2.5 promotes IL-13 expression through the activation of the JAK2/STAT6 pathway, which leads to further airway inflammation [39]. Similarly, Ho et al. showed that prolonged exposure to PM (for a total of 6 weeks) led to increases in both M1 and M2 cytokines. In addition, higher M2 cytokine levels were found in the PM2.5 group, whereas higher M1 cytokine levels were noted in the coarse PM group, which implies that small particle PM may be more potent to the allergic response [40]. In conclusion, the current evidence suggests that exposure to PM initially induces a predominantly proinflammatory response to eliminate the foreign threat, but prolonged exposure may also promote allergic responses that contribute to chronic lung disorders such as asthma.

## 7. Effects of Heavy Metals on Macrophage Polarization

The composition of PM is complicated. It mostly contains elemental and organic carbons, heavy metals, sulfates with nitrate, and other components [80]. Earlier immunotoxicology studies focused on the influence of PM on health, as it was considered the most important component in air pollutants. However, other components in the PM may also participate in this adverse effect. It has been found that coexposure to particulate-depleted diesel exhaust and allergens dampened lung function via increased NO_2_ despite the absence of the particulate component [5,93]. There is growing evidence that heavy metals adsorbed to PM are crucial to the toxicity and adverse health effects of PM [94,95]. Heavy metals, as significant chemical components in PM, come from either natural sources or human sources such as industrial production, residential heating, automobile exhaust emissions, and so on [96]. The WHO published guidelines for air quality in 2000 that reported that the major heavy metals in PM included lead (Pb), vanadium (V), arsenic (As), manganese (Mn), nickel (Ni), cadmium (Cd), and chromium (Cr(VI)) [97]. In this review, we focus on effects of Pb, Cd, As, Ni, and Cr (VI) on macrophage polarization. The alterations of macrophage-related cytokines after heavy metal exposure are shown in Table 2.

### 7.1. Lead (Pb)

Lead (Pb) is found naturally in the environment as well as in manufactured products. According to the U.S. Environmental Protection Agency, the major sources of lead in the air are ore and metal processing and piston-engine aircraft operating on leaded aviation fuel. From 1980 to 2014, the concentration of Pb in the air decreased by about 98% due to one reason: the removal of lead from motor vehicle gasoline. Because of the waste incinerators, utilities, and lead–acid battery manufacturers, lead is still an air pollutant [117]. Previously, studies suggested that Pb could result in impaired macrophage functions, including decreased chemotactic activity and phagocytosis ability in animal studies [101]. Recently research has indicated that 5 or 10 μg/dL Pb can increase the inflammation-related genes COX-1 and COX-2 and the concentrations of thromboxane B2, prostaglandin E2 (PGE-2), and proinflammatory cytokines (IL-1β and IL-6) in the human monocyte cell line THP-1 [98]. In a mouse study involving exposure to environmentally relevant concentrations of Pb (6 μg/dL), bone-marrow-derived macrophages (BMDM) were used to investigate the effects of Pb on inflammation and anti-inflammation. They found that Pb could reduce NO production and increase major histocompatibility complex class II (MHC II) expression but could not affect the production of the proinflammatory cytokines TNF-α and IL-1β or CD86 expression in LPS/IFN-γ stimulated BMDM. In contrast, Pb increased TGF-β1 and mannose receptor (CD206) expression in IL4/IL-13-stimulated BMDM. This might explain the susceptibility to allergic diseases reported in subjects exposed to Pb [99]. Another study used THP-1-derived macrophages to evaluate the cellular effects of Pb nanoparticles. They showed that Pb decreased mitochondrial activity and ROS production but increased LDH release and the production of the proinflammatory cytokine IL-8. They also indicated that Pb nanoparticles were stronger immune inducers than a Pb(NO_3_)_2_ solution. These results suggest that the inhalation of Pb nanoparticles may cause pulmonary inflammation [100].

### 7.2. Vanadium (V)

Vanadium (V) a widely distributed and fairly abundant element in the Earth’s crust and is commonly used to manufacture tools, orthopedic implants, and machinery due to its hardness, ability to form alloys, and high resistance to corrosion. The total emission of V is estimated to be between 71,000 and 210,000 tons per year worldwide [118]. In a previous study, the authors used 5 μg/mL sodium metavanadate (NaVO_3_) to observe the inflammatory effect in the RAW 264.7 macrophage cell line. The results showed that NaVO_3_ could increase CXC chemokine macrophage inflammatory protein-2 (MIP-2/CXCL-2) mRNA expression in a dose-dependent manner, and the expression could be suppressed by an antioxidant N-acetylcysteine treatment. These results indicated that NaVO_3_ induced CXCL-2 mRNA expression via increased oxidative stress [102]. Cohen et al. studied the effect of ammonium metavanadate (NH_4_VO_3_) and vanadium pentoxide (V_2_O_3_) on murine macrophage-like cells. The results indicated that V suppressed TNF-α production but increased prostaglandin E2 (PGE2) production [103]. The other study by Cohen et al. used the V-exposed rats to examine the inflammation effects. After 2 mg V/m^3^ (as NH_4_VO_3_) for 8 h/d for 4 d, the rats received an intratracheal (it) instillation of polyinosinic.polycytidilic acid (poly I:C) or saline, and bronchoalveolar lavage fluid (BALF) was collected for cytokine determination. The levels of IL-6 and IFN-γ in BALF were increased by V exposure alone, but TNF-α and IL-1 were not affected. The BALF IL-6 and IFN-γ levels were significantly increased by the poly l:C induced in the poly l:C and V coexposure groups. However, V-exposed rats produced significantly less cytokines when the cytokine levels were calculated with total lavaged protein [104].

### 7.3. Arsenic (As)

Arsenic (As) is generally released into the environment from various natural sources such as volcanic eruptions and forest fires. Several studies have been published on the health effects of arsenic in water. However, more As is released into the atmosphere due to human activities such as coal burning and industrial waste disposal with industrial development [119]. Therefore, it is suggested that atmospheric As is a high-ranking health risk to the respiratory system.

In As-exposed (0.5 mg/kg/body weight sodium arsenite solution) Swiss albino mice, researchers gave an intravenous injection of *Staphylococcus aureus* and collected and cultured blood at different intervals after injection. Compared with the control group, few bacteria from the blood of As-exposed mice were still cultured, which showed slower clearance. In addition, the splenic macrophages from As-exposed mice showed reduced adhesion and migration index values [120]. After As stimulation, the adherent ability and surface marker CD86 were reduced [107], but the amount of apoptosis/necrosis in primary human monocyte-derived macrophages increased [105]. The production of NO, IL-1α, IL-8, IL-12, and TNF-α in As-induced primary human monocyte-derived macrophages was markedly higher than in the untreated group [105,107]. Emilie Bourdonnay et al. reported that As could increase CCL-22 and CXCL2 in human primary monocyte-derived macrophages, which was reversed by an N-acetylcysteine treatment. This demonstrated that arsenic alters macrophage gene expression through redox-sensitive signaling pathways [108,109]. Another study revealed that As could increase M2 markers and CD206 and CD163 expression in THP-1-derived M2 macrophages. In addition, IL-6, IL-10, TGF-β, and CCL-18 were significantly increased in As-induced THP-1-derived M2 macrophages [106].

### 7.4. Manganese (Mn)

Manganese (Mn), an essential trace element in humans, is naturally present in many foods and is available as a dietary supplement. However, industrial processes such as welding, mining, smelting, battery manufacturing, and steel production can lead to high Mn release into the environment from dust particles containing Mn [121].

Matlou I. Mokgobu et al. used primary human monocyte-derived macrophages to investigate the effects of MnCl_2_ on immune responses as a potential mechanism of MnCl_2_ toxicity. They found that 100 μM MnCl_2_ could significantly increase the inflammatory cytokines IL-1β, IL-6, IL-8, TNF-α, and IFN-γ and intracellular H_2_O_2_ production in primary human monocyte-derived macrophages. MnCl_2_-induced IL-6 and IL-8 were partially inhibited by p38 MAPK and NF-κB inhibitors but were abolished entirely by dithiothreitol ROS scavenger treatment. This suggested that MnCl_2_ causes increased primary monocyte-derived macrophage inflammatory cytokine production through oxidative stress activation [110].

### 7.5. Nickel (Ni)

Nickel (Ni), one of the ferromagnetic elements, is naturally present in the Earth’s crust. Due to its unique physical and chemical properties, Ni is used in a wide variety of metallurgical processes such as alloy production, electroplating, batteries, and as a catalyst in the chemical and food industries. In all stages of manufacturing, recycling, and disposal, Ni unavoidably causes pollution in the environment [122].

In a previous study, researchers found that lung inflammation and fibrosis were significantly increased, but the counts of macrophages in bronchoalveolar lavage fluid (BALF) from Ni-nanoparticle-exposed mice were significantly decreased after 21 days of exposure. The CCL-2 concentration in BALF was significantly increased in Ni-nanoparticle-exposed mice [112]. In contrast, Katherine A. Roach et al. used fine NiO particles (NiO-F, 181 nm) and ultrafine particles (NiO-UF, 42 nm) to expose BALB/cJ mice and evaluated the effect of NiO on allergies. The lactate dehydrogenase, total cells, and levels of IL-6, IFN-γ, and TNF-α in BALF from NiO-UF-exposed mice were significantly increased compared to the NiO-F-exposed or control groups after 10 days of NiO exposure. In NiO-UF-exposed mice with OVA challenge, the levels of IFN-γ and IL-2 in both BALF and serum, IL-6 in BALF, and IL-12p40 in serum were significantly increased compared to the OVA control groups. These findings demonstrated that the NiO surface area correlates best with acute pulmonary injury and inflammation following respiratory exposure [111].

### 7.6. Cadmium (Cd)

Cadmium (Cd), a metal belonging to group IIB of the periodic table, is emitted into the environment due to natural activities such as volcanic activity. However, anthropogenic activities also release Cd pollution into the environment, including tobacco smoking, the combustion of fossil fuels, waste incineration, and releases from tailings piles and municipal landfills [123]. Marit Låg et al. used cadmium acetate to treat rat alveolar macrophages and evaluated the effect of Cd-induced lung toxicity. In low concentrations of Cd (3 μM) treatment, the expression of IL-1β, IL-6, and TNF-α mRNA was significantly decreased after 10 h of stimulation. However, the protein production of MIP-2, IL-1β, and TNF-α was significantly increased by 10 μM Cd treatment for 20 h, but the levels of IL-6 were not affected by the different doses of Cd (1, 3, 6, and 10 μM) [113]. In another study, they used a human monocyte cell line, THP-1, and THP-1-derived macrophages to investigate the effect of Cd on macrophage immune function. They found that Cd significantly increased IL-6 and TNF-α production in THP-1 cells but significantly decreased IL-6, IL-8, and TNF-α production in THP-1-derived macrophages. In Cd-exposed mice, BALF macrophages were significantly increased compared to the control group. However, the levels of IL-6, CXCL-2, and TNF-α in lipopolysaccharide-induced BALF were significantly decreased in Cd-exposed mice compared to the control group. They also found that Cd suppressed M1-related cytokine gene expression, including CXCL-9, CXCL-10, CXCL-11, and CCL-5. These results revealed that Cd exposure prior to an endotoxin challenge impaired the capacity of macrophages to elicit a proper immune response [114].

### 7.7. Chromium (Cr(VI))

Chromium (Cr) is the 17th most abundant element in the Earth’s mantle and is formed naturally as chromite in ultramafic and serpentine rocks. However, the environmental contamination of Cr has increased in recent years due to it being widely used in industry for plating, alloying, the tanning of animal hides, the inhibition of water corrosion, textile dyes and mordants, pigments, ceramic glazes, and refractory bricks.

Induced nitric oxide (NO) production is an important function of macrophages. Tian and Lawrence studied the effects of different metals, including chromium, and reported that chromium does not affect NO production by cytokine-stimulated (IFN-γ and TNF-α) murine macrophages. Chromium also showed a suppressed effect on inducible NO synthase expression, which suggests that it may directly modify enzyme or cofactor activity [124]. In addition to the impaired production of NO, an in vitro experiment showed that Cr also impaired the phagocytosis of bovine macrophages [125]. Jain and Kannan reported that the decreased TNF-α secretion in U937 monocytes due to chromium stimulation was mediated by its antioxidative effect [115]. The exposure of urban women to chromium from vehicular traffic affects their immune function. Serum IgE and the production of IL-4 and IFN-γ by mononuclear blood cells were increased in urban women who were exposed to vehicular traffic [116]. Several studies have investigated the effects of prostheses on the cytokine responses of the body. A previous study examined the effects of CoCr alloy particles on basic biological responses, including cell proliferation, apoptosis, cytokine mRNA expression, and protein secretion, in the J774A.1 murine macrophage cell line. The results indicated that the levels of TNF-α, IL-1α, IL-6, and IL-12 in the supernatant were not significantly different between the control group and cells stimulated with CoCr. A change in the metabolic activity of J774A.1 cells was only observed with higher concentrations of CoCr particles [126].

## 8. Conclusions

**Figure 1:**
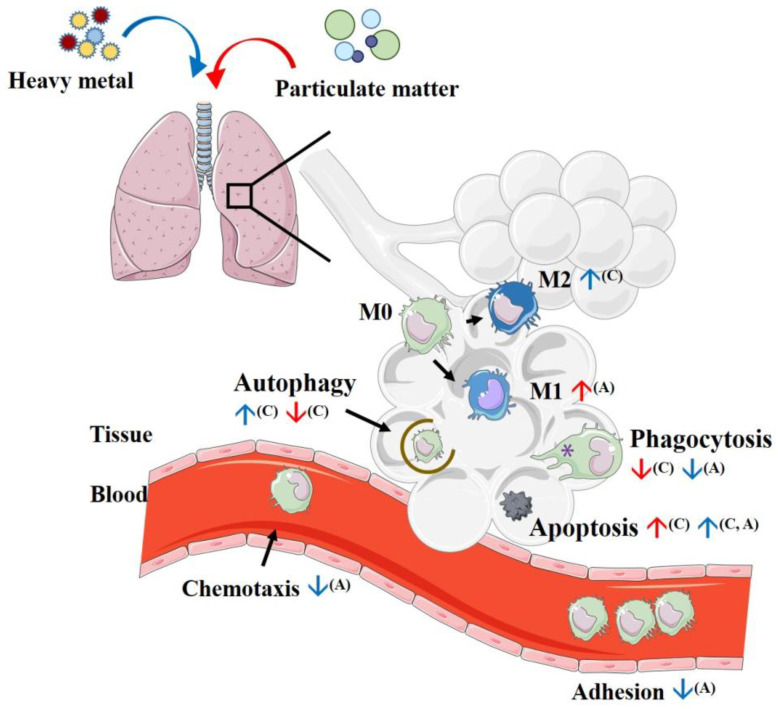
Alteration of macrophage function after air pollutant exposure. After particulate matter exposure (red arrow), the macrophages in the bronchial alveolar lavage fluid increased with PM2.5 exposure in OVA asthma models. However, their functions, including phagocytic activity and autophagy, were decreased, and cell apoptosis increased. After exposure to heavy metals (blue arrow), the macrophages differentiated into M2 macrophage profiles. The functions of macrophages, including phagocytic activity, chemotaxis, and adhesion ability, also decreased, and cell autophagy and apoptosis increased. ^(A)^ indicates experimental results from animal models and ^(C)^ indicates experimental results from cell models. The figure was partly generated using Servier Medical Art, provided by Servier, licensed under a Creative Commons Attribution 3.0 unported license.

PM is a major pollutant in air pollution that mostly contains elemental heavy metals. In summary, PM and heavy metal exposure to alveolar macrophages initially promotes the production of proinflammatory cytokines to clear harmful stimuli by local inflammation. However, persistent PM and heavy metal exposure lead to macrophage dysfunction (Figure 1), including decreased phagocytosis, decreased efferocytosis, and increased apoptosis; these effects also increase the vulnerability of the respiratory tract. Prolonged PM and heavy metal exposure also promotes airway M2 cytokine production, which leads to further allergic responses in the respiratory tract. Furthermore, the airway hyper-responsiveness induced by PM exposure was demonstrated in a recent study; exposure to Afghanistan PM in mice resulted in suppressed expression of a regulator of G-protein signaling 2 (RSG 2, a gene known to protect against bronchoconstriction) [127]. Taken into consideration, alveolar macrophages have an important role in the development of the lung inflammation induced by exposure to PM and heavy metals in in vitro studies. Further investigation of the underlying immunological mechanism in in vivo and gene–environmental interaction research may provide a new approach to reducing the toxic effects of PM and heavy metals in developing chronic inflammatory disorders such as asthma.

## Figures and Tables

**Table 1:** The changes in macrophage-related cytokines and inflammatory mediators after exposure to particulate matter.

Pollutants	M1 Phenotype	M2 Phenotype
ultrafine particles (UFP)	Leukotriene B4 ↑ ^(A)^ [37]	Prostaglandin E2 (PGE-2) ↑ ^(A)^ [37]
	8-isoprostane ↑ ^(A)^ [37]	15(S)-HETE ↑ ^(A)^ [37]
		Lipoxin A4 ↑ ^(A)^ [37]
PM 2.5	IL-1β ↑ ^(H)^ [38], ↑ ^(A)^ [39,40]	IL-10 ↑ ^(A)^ [40,41], ↓ ^(A)^ [42]
	IL-6 ↑ ^(H)^ [38,43], ↑ ^(A)^ [40,44]	Prostaglandin D2 ↑ ^(A)^ [39]
	IL-8 ↑ ^(H)^ [38,43]	PGE-2 ↑ ^(C)^ [45]
	IL-12 ↑ ^(A)^ [39,40]	Arginase ↓ ^(C)^ [42]
	TNF-α↑ ^(H)^ [38,43], ↑ ^(A)^ [41,42,44,46]	
	IFN-γ ↑ ^(A)^ [47], ↓ ^(A)^ [41]	
	CCL-2 ↑ ^(A)^ [39,44]	
	CCL-3 ↑ ^(A)^ [40]	
	iNOS ↑ ^(A)^ [40,42]	
PM 10	IL-1β ↑ ^(H)^ [48], ↑ ^(A)^ [40,49]	IL-10 ↑ ^(H)^ [50], ↑ ^(A)^ [40]
	IL-6 ↑ ^(H)^ [51], ↑ ^(A)^ [52]	PGE-2 ↑ ^(A)^ [52]
	IL-8 ↑ ^(H)^ [50,51]	
	IL-12 ↑ ^(A)^ [40,52]	
	TNF-α ↑ ^(H)^ [50], ↑ ^(A)^ [40,46,52]	
	CCL-2 ↑ ^(A)^ [40]	
	CCL-3 ↑ ^(A)^ [40]	

Note: ^(A)^ indicates experimental results from animal models; ^(C)^ indicates experimental results from cell models; and ^(H)^ indicates experimental results from human subjects; ↑ indicates increased the level of cytokines or mediators; ↓ indicates decreased the level of cytokines or mediators.

**Table 2:** The changes in macrophage-related cytokines and surface markers after exposure to heavy metals.

Pollutants	M1 Phenotype	M2 Phenotype
Lead (Pb)	IL-1β ↑ ^(C)^ [98]	CD206 ↑ ^(A)^ [99]
	IL-6 ↑ ^(C)^ [98]	TGF-β1 ↑ ^(A)^ [99]
	MHC class II ↑ ^(A)^ [99]	
	IL-8 ↑ ^(C)^ [100]	
	IFN-γ ↓ ^(C,A)^ [101]	
	IL-12p40 ↓ ^(A)^ [101]	
Vanadium (V)	CXCL-2 ↑ ^(C)^ [102]	
	TNF-α ↓ ^(C)^ [103]	
	IL-6 ↑ ^(A)^ [104]	
	IFN-γ ↑ ^(A)^ [104]	
Arsenic (As)	TNF-α ↑ ^(C)^ [105]	CD206 ↑ ^(C)^ [106]
	IL-1α ↑ ^(C)^ [107]	CD163 ↑ ^(C)^ [106]
	IL-8 ↑ ^(C)^ [105]	IL-10 ↑ ^(C)^ [106]
	IL-12 ↑ ^(C)^ [107]	CCL-18 ↑ ^(C)^ [106]
	CXCL2 ↑ ^(C)^ [108,109]	TGF-β1 ↑ ^(C)^ [106]
	CD86 ↓ ^(C)^ [107]	CCL-22 ↑ ^(C)^ [108,109]
Manganese (Mn)	IL-1β ↑ ^(C)^ [110]	
	IL-6 ↑ ^(C)^ [110]	
	IL-8 ↑ ^(C)^ [110]	
	TNF-α ↑ ^(C)^ [110]	
	IFN-γ ↑ ^(C)^ [110]	
Nickel (Ni)	IL-2 ↑ ^(A)^ [111]	
	IL-6 ↑ ^(A)^ [111]	
	IL-12 ↑ ^(A)^ [111]	
	IFN-γ ↑ ^(A)^ [111]	
	TNF-α ↑ ^(A)^ [111]	
	CCL-2 ↑ ^(A)^ [112]	
Cadmium (Cd)	IL-1β ↑ ^(C)^ [113]	
	TNF-α ↑ ^(C)^ [113], ↓ ^(C,A)^ [114]	
	CXCL2 ↑ ^(C)^ [113], ↓ ^(A)^ [114]	
	IL-6 ↓ ^(C,A)^ [114]	
	IL-8 ↓ ^(C)^ [114]	
	CXCL-9 ↓ ^(C)^ [114]	
	CXCL-10 ↓ ^(C)^ [114]	
	CXCL-11 ↓ ^(C)^ [114]	
	CCL-5 ↓ ^(C)^ [114]	
Chromium (Cr)(VI)	TNF-α ↓ ^(C)^ [115]	
	IFN-γ ↑ ^(H^^)^ [116]	

Note: ^(A)^ indicates experimental results from animal models; ^(C)^ indicates experimental results from cell models; and ^(H)^ indicates experimental results from human subjects; ↑ indicates increased the level of cytokines or mediators; ↓ indicates decreased the level of cytokines or mediators.
